# Shop Talk: Annual *Drosophila* Research Conference, 2010

**DOI:** 10.1002/dvdy.22422

**Published:** 2010-11

**Authors:** Gerald B Call, Oorvashi Roy Puli, Shilpi Verghese, Amit Singh

**Affiliations:** 1 Department of Pharmacology, Midwestern UniversityGlendale, Arizona; 2 Department of Biology, University of DaytonDayton, Ohio; 3 Center for Tissue Regeneration and Engineering at Dayton (TREND)Dayton, Ohio

**Keywords:** *Drosophila melanogaster*, patterning, development, *Drosophila* eye, Genetics Society of America, Fly meeting

## INTRODUCTION

**Fig. 1:**
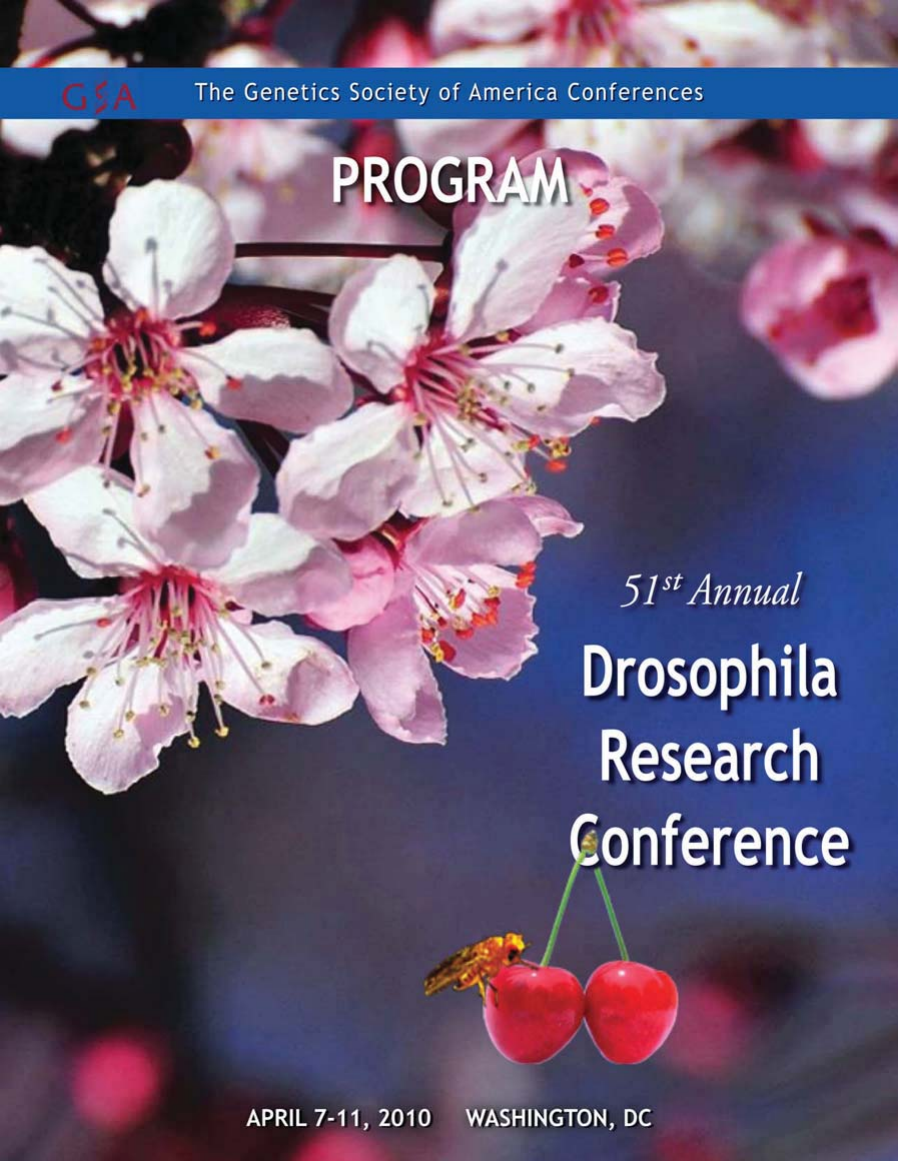
Cover page of the abstract book of the 51st Annual *Drosophila* Research Conference held at Washington DC from April 7–11, 2010. Courtesy: Genetics Society of America (GSA).

The 51st Annual *Drosophila* Research Conference took place in Washington, DC, from April 7–11, 2010. Washington, DC, the U.S. capital, is known for its national buildings and historically significant monuments. The Cherry Blossom festival, which was one of the major tourist attractions of the week, was at its peak – as can be seen on the program cover (Fig. 1). This year, the Genetics Society of America (GSA) received a record-breaking number of registrants to the conference. Despite low attendance at other scientific meetings, according to GSA Meetings Manager, Suzy Brown, this year's conference had the “largest number of registrants than any other previous years.” There were 170 talks, more than 850 posters and 13 workshops; so there was a range of information that people could pick according to their interests. Two members of the *Drosophila* community received GSA honors. Sherry Marts, GSA executive director, awarded Utpal Banerjee (University of California, Los Angeles) the Elizabeth W. Jones Award for Excellence in Education and William Gelbart (Harvard University, Cambridge) received the George W. Beadle Award for outstanding contributions to the community of genetics researchers. The winner of the Larry Sandler Memorial Lecture Award was Leonardo Barbosa Koerich, a student of Antonio Carvalho (Universidade Federal do Rio de Janeiro, Brazil). The topic of his dissertation was the low conservation of gene content of the Y chromosome across 12 species of *Drosophila*. He worked with 11 genes from the *Drosophila melanogaster* Y chromosome and located them on the other sequenced *Drosophila* species. His work was a very nice evolutionary genetic analysis revealing that the Y chromosome of *Drosophila melanogaster* is actually gaining more genes than losing. This goes against the canonical theory that the Y chromosome is a degenerated X-chromosome that will eventually become fully degenerated.

Leonardo's seminar was followed by a panel presentation with a theme discussing the major achievements of renowned *Drosophila* scientists and their impact on the direction of *Drosophila* research. The panel was introduced by Hugo J. Bellen (Baylor College of Medicine, Houston) and was started by Thomas Kaufman (Indiana University, Bloomington), who talked about the discovery of the *white* (*w*) gene and how it led to future *Drosophila* genetics. Gerald M. Rubin (Janelia Farm, HHMI, Ashburn) discussed the cloning of *w* and the sequencing of the genome. Allan Spradling (Carnegie Institution for Science, Baltimore) talked about P-elements and their use in transformation, insertional mutagenesis, and the Genome Disruption Project. Susan Celniker (University of California, Berkley) gave a history of the genome project after the initial sequencing was completed and the development of more genomic tools, including the very high-throughput next generation sequencers. Norbert Perrimon (Harvard Medical School, Boston) discussed how the sequencing of the genome has impacted new tool development for *Drosophila melanogaster*. Finally, William Gelbart talked about annotation of the *Drosophila* genome then and now, and delivered a brief history of FlyBase.

The First Plenary Session began with the award ceremony of the Annual *Drosophila* Image Award. Among the 50 images submitted for this award, only 10 were selected as finalists. Guy Blanchard (University of Cambridge, UK) won the award for an outstanding image demonstrating quantitative cell shape changes during gastrulation. Images from F. Coumailleau et al. (University of Geneva, Switzerland) showing directional transport of endocytosed Delta during asymmetric cell division and S. MacArthur et al. (Lawrence Berkeley National Laboratory, Berkeley) demonstrating quantitative differences in DNA occupancy by transcription factors that determine very different output expression patterns were runners-up for the image award.

Duojia (DJ) Pan (Johns Hopkins University School of Medicine, Baltimore, MD) started off the first plenary session talks discussing his work in the control of organ size and tumorigenesis by the Hippo (Hpo) pathway. He showed how the Hpo kinase cascade regulates the gene cascade causing apoptosis and proliferation. Previous work from DJ's laboratory has demonstrated that the mammalian pathway is highly conserved and functions in mice as it does in *Drosophila melanogaster*. Over-expression of a downstream component of the Hpo pathway, YAP [the mammalian homolog of *yorkie* (*yki*)], in the mouse liver causes a reversible increase in liver size; but chronic over-expression leads to hepatocellular carcinoma. Kenneth D. Irvine (Rutgers, The State University of New Jersey, Piscataway) talked about his work on Fat (Ft) as a tumor suppressor and a regulator of planar cell polarity. Ft is a cadherin molecule and the Ft pathway has distinct mechanisms that regulate growth versus planar cell polarity. He showed that Ft is part of the Dachs (D), Dachsous (Ds), and Four-jointed (Fj) pathway. Fj and Ds are expressed in complementary gradients. Ds is a ligand that activates Ft, but also polarizes the cells. Clones of *ds* and *fj* have opposite effects on planar cell polarity, but have the same transcriptional effects. Chiara Cirelli (University of Wisconsin, Madison) discussed her work on sleep and synaptic plasticity. It is thought that sleep does one of two things in the CNS: enables synaptic plasticity or glycogen replenishment. The glycogen theory is not supported in all animals. Interestingly, fly sleep is very similar to mammalian sleep. In sleep-deprived flies, the expression of synaptic proteins is increased in whole brains and single neurons, giving support to the synaptic plasticity theory. Interestingly, similar changes were seen in *period* (*per*) mutants that have circadian rhythm problems.

Ting Xie (Stowers Institute for Medical Research, Kansas City, MO) started off the next part of the first plenary session by talking about his contribution to the understanding of germline stem cells (GSC) in the *Drosophila* ovary. He highlighted the concept that GSCs have a collaborative and rival relationship with one another. For example, he identified some mutant GSCs that have a more competitive advantage over normal stem cells and actually push the wild-type stem cell out of its niche. These mutant cells express more E-cadherin than the wild-type GSC, so it is retained in the niche better. He also discussed his findings of how apparently routine “housekeeping” genes can also be specifically involved in the maintenance of GSCs. These general proteins, including eIF4A and CSN4, have GSC phenotypes, while other family members of these proteins do not have GSC phenotypes. Eric H. Baehrecke (University of Massachusetts Medical School, Worcester) discussed his work on macro-autophagy, which is considered a bulk, non-selective method of destruction. Autophagy is involved in the death of at least four different cell types in *Drosophila*. The model he uses is salivary gland cells, since at 12 hr after puparium formation they undergo autophagy. He demonstrated that caspases or autophagy genes are sufficient to kill salivary gland cells and they act in an additive manner. He found that some of the engulfment genes that were turned on in his microarray analysis were phagocytosis receptors, including *draper* (*drpr*). By expressing *drpr* RNAi in the salivary gland, autophagy is inhibited. However, *drpr* appears to have no role in autophagy in the blood or fat body (other sites of autophagy in *Drosophila*). Lyn Cooley (Yale School of Medicine, New Haven, CT) discussed her work on oogenesis. She found a reversible response to nutritional stress in the post-germarium to pre-vitellogenic stages of oogenesis. She performed an oocyte-enriched protein trap screen and found many RNA-binding proteins that localize to the oocyte through a microtubule-dependent mechanism. In the protein-poor condition (i.e., nutritional stress), many of these RNA-binding proteins start forming aggregates in the nurse cells, which might be due to microtubular condensation. This process is reversible; by giving the females food again, the aggregates disappear. She found that this nutrition response works through insulin signaling: reduced egg chamber production in poor food conditions operates in an insulin-dependent manner. Apart from the excellent first plenary session, the conference was composed of multiple platform and poster sessions on many topics. Since we are not able to cover the broad spectrum of topics presented in this meeting, we present some highlights in this report.

## STEM CELLS

*Drosophila melanogaster* has become a premier model for studying stem cells in an ever-increasing variety of tissues. Guonan Lin (National Institute for Biological Sciences, Beijing, China) is interested in determining how the intestinal stem cell (ISC) is maintained. Published work identified that Wingless (Wg) signaling from the surrounding muscle cells maintains and controls self-renewal of the ISCs. More recent work from their laboratory demonstrated that Unpaired (Upd), a ligand in the JAK/STAT (JAK, Janus kinase; STAT, signal transducers and activators of transcription) pathway in *Drosophila*, is expressed in the ISC niche as well. STAT signaling is active in ISCs and other cell types around the ISC niche. Clonal analysis indicates that Upd is necessary for ISC maintenance and proliferation and that *hopscotch* (*hop*) and *domeless* (*dome*) (other components of the JAK/STAT pathway) mutants have ISC loss, which suggests that it is important for maintenance and lineage differentiation. Judy Leatherman (University of Pennsylvania, Philadelphia) reminded us that in the past their lab had shown that there was a second signal from the cyst stem cell, in addition to the GSC-specific JAK/STAT pathway, which allowed the GSC in the testis to maintain self-renewal. They knocked down STAT in all tissues and simultaneously over-expressed STAT in the cyst cells. Interestingly, this model of germline-specific STAT loss did not have germline loss like global STAT loss produces. In fact, the STAT-deficient GSCs survive and appear to differentiate normally. Therefore, the STAT signal in the cyst cells is promoting GSC maintenance through some secondary signal, reinforcing the idea of a secondary signal. Justin Voog (Salk Institute for Biological Studies, La Jolla, CA) also works on male GSCs. Mutants of *escargot* (*esg*) have hub cell loss, which are the male GSC niche; thus, the germline is lost as well in *esg* mutants. He asked the question if Esg is sufficient to maintain hub cells. Over-expression of *esg* induces ectopic somatic cells that express hub markers as well as more germ cells that are STAT- and phospho-Smad-positive, which suggests the presence of more GSCs. Since Esg is a transcription factor, they performed a DamID experiment to identify transcriptional targets of Esg. They identified *stat92e* and *Zfh-1* in this experiment. Further experiments demonstrated that these genes are required or can modify the cell's response to *esg* expression.

## CELL BIOLOGY AND GROWTH

Ekin Bolukbasi (University of Edinburgh, Edinburgh, UK) discussed the role of the gene *poly*. Mutation of *poly* results in pleiotropic effects, including smaller larval brains and absent or reduced imaginal discs. Poly levels are decreased in *Insulin-like receptor* (*InR*) mutant larvae and *poly* mutation results in decreased signaling activity downstream of InR. Interestingly, autophagy is constitutively active in *poly* mutants, as it is present in the fat bodies of well-fed larvae, where autophagy normally does not occur. Staining for Poly in both hemocytes and HeLa cells is enhanced following insulin stimulation. This data, along with microarray analysis, suggest that Poly is a novel regulator of cell growth and metabolism. Shizue Ohsawa (Kobe University, Japan) is interested in understanding how normal epithelial cells surrounding a group of polarity-defective cells can remove and recover for those mutant cells. It has been previously demonstrated that polarity-deficient epithelia overgrow, as seen in *scribble* (*scrib*) mutant imaginal discs. Interestingly, clones of *scrib* mutant cells are eliminated in eye imaginal discs if there are wild-type cells present. However, if the normal cells are eliminated, the *scrib* mutant cells are not removed. Therefore, some mechanism within the normal cells must be present that allows the surrounding wild-type cells to remove and recover for the mutant cells. They found that Jun N-terminal kinase (JNK) is activated in both polarity-deficient and surrounding wild-type cells. This JNK activation in the surrounding wild-type cell does not induce cell death; however, it does promote elimination of the *scrib* mutant clones. They found that JNK signaling works via PDGF- and VEGF-receptor related (PVR) to do this. PVR is up-regulated in mutant and surrounding wild-type cells. By eliminating or over-expressing PVR in *scrib* clones, you can rescue or enhance cell death, respectively. Adrian Halme (University of Virginia, Charlottesville) studies regenerative growth and developmental progression. Previous studies have demonstrated that transplantation of damaged imaginal discs in larvae can slow down the development of the host larvae. This developmental delay can also occur by irradiating larvae as well. However, late in the third instar there is a restriction point after which this developmental delay will no longer occur. Prothoracicotropic hormone (PTTH) expression in the larval brain drives expression of ecdysone during development to cause molting. Interestingly, the expression of PTTH is significantly delayed in irradiated animals or in animals with tissue damage. Constitutive expression of PTTH in animals with tissue damage overcomes this delay. They found that JNK is a dominant modifier of this process and that mutations in the JNK pathway can also regulate this developmental delay. Helena Richardson (Peter MacCallum Cancer Center, Melbourne, Australia) works on the oncogenic co-operation of Scrib and activated Ras in the manifestation of neoplastic tumors. Scrib alone causes hyperplastic growth; however, when it interacts with activated Ras it causes invasive tumors. They proposed that RhoGEF2 and activated Ras interact with each other to up-regulate JNK signaling, in addition to giving an invasive phenotype, similar to the Scrib and Ras phenotype. Brian S. Robinson (Emory University School of Medicine, Atlanta, GA) works on the role of Crumbs (Crb) in the Salvador-Warts-Hippo signaling cascade. Crb-dependent growth is mediated through Yki. When the intracellular domain of Crb is expressed, Yki and Drosophila inhibitor of apoptosis (DIAP1)-LacZ levels are elevated and Expanded (Ex) levels are reduced. Interestingly, Ex is mislocalized in *crb* mutants. Woulter Bossuyt (MD Anderson Cancer Center, Houston, TX) discussed the role of F-Actin in Hpo signaling. They found that increased F-Actin activates Hpo pathway components. Thus, proper actin dynamics is important for normal growth control. Molly Duman-Scheel (Indiana University School of Medicine, South Bend) presented her work on the role of a netrin receptor, *frazzled* (*fra*), as a tumor suppressor gene. This gene encodes for the *Drosophila* ortholog of deleted in colorectal cancer (DCC). Frazzled/DCC is a metastatic tumor suppressor gene whose loss is characterized by E-cadherin mislocalization, reorganization of the actin cytoskeleton, loss of apico-basal polarity, over-proliferation, metastasis, and increased MAPK signaling whereas Frazzled/DCC over-expression is characterized by overgrowth without metastasis.

## PHYSIOLOGY AND AGING

The *Drosophila* model, with a wide array of genetic tools, has become an increasingly popular organism to study physiological processes and to determine any genetic influences or determinants involved. Tania Reis (University of California, Berkley) performed a screen for fat larvae by identifying floating larvae in liquid. She identified *Sir2* in the screen and found its expression in all issues (fat body, salivary gland, and so on). Interestingly, RNAi knockdown of *Sir2* in the fat body and neurons reduced overall body fat. After other experiments, she concluded that Sir2 might function as a possible nutritional sensor. Marc Tatar (Brown University, Providence, RI) discussed work performed on juvenile hormone, a steroid hormone produced by the corpora allata, which has been known for over 50 years but is still an orphan ligand. Juvenile hormone extends life in other insects, as determined by corpora allata removal. Interestingly, lifespan in *chico* and *InR* mutants is longer as well, and administration of juvenile hormone in these mutants restores lifespan back to normal. By using the UAS/GAL4 system, he was able to genetically allotectimize (CAX) *Drosophila*. This resulted in reduced fecundity and increased lifespan. CAX animals have increased stress resistance to starvation and peroxide challenge. Jason Tennessen (University of Utah, Salt Lake City) reported that the genome of *Drosophila melanogaster* encodes 18 canonical nuclear receptors, which includes all 6 mammalian classes. Mutations in the *Drosophila estrogen-related receptor* (*dERR*) die abrubtly in the second instar. Metabolic analysis indicates that *dERR* mutants have low ATP levels, even though they have high levels of circulating sugars and normal glycogen. ERRα has been shown to be a global regulator of energy metabolism in mice. After much metabolic profiling, it appears that dERR promotes aerobic glycolysis, which increases glycolysis, lactate production, pentose phosphate shunt, and proline/glutamine production. This is similar to the Warburg effect for energy production, which is utilized by rapidly-growing cancer cells. This is possibly the first documented developmental use of the Warburg effect, which could be a mechanism of energy production that would allow for exponential larval growth. Reinhard Bauer (University of Bonn, Germany) determined that P-element insertion mutants in the ceramide synthase, *schlank*, have decreased growth. Larvae that are mutant for *schlank* are thinner and more transparent. Triglycerides are decreased in *schlank* mutants or in larvae that express *schlank* RNAi, while they are increased with Schlank over-expression. Schlank is required in the fat body and negatively regulates lipolysis. He proposed that Schlank might be a regulator of the balance between lipogenesis and lipolysis. Lauren Killip (University of Calgary, Canada) is interested in identifying the downstream effectors below Target of rapamycin (Tor) that can cause a Tor-like effect on a cell's ribosome biogenesis. Starved larvae have decreased nucleolar size, but over-expression of Rheb will increase nucleolar size. Microarray analysis of fed versus starved larvae identified a group of ribosome biogenesis genes being responsive to nutrition. These genes all shared a DNA replication-related element factor (Dref) site. Dref knockdown reduces cell and larval growth, and most were lethal in a cell-autonomous manner. Reduced Dref leads to decreased mRNA for ribosome genes. Reduced nutrition or Tor signaling exacerbates Dref loss-of-function phenotypes in both eye and wing discs. This suggests an interaction between Dref and the Tor pathway. Starved larvae and cells inhibited by rapamycin (which inhibits Tor) showed decreased Dref. So Dref is involved in the transcriptional process of ribosome biogenesis and may be downstream of the insulin/Tor pathway.

## EYE DEVELOPMENT

Because of the redundant and simple model of the *Drosophila* eye, it has long been a favorite system for studying eye development. Robert J. Johnston (New York University) discussed the expressivity of the different Rhodopsins (Rh), which play a major role in the formation of the photoreceptors in the eye. The *Drosophila* eye is made up of outer and inner photoreceptors (R1–R8 cells). These photoreceptors undergo differentiation, which initiates Rh expression for the formation of rhabdomeres. It is already known that R1–R6 are the outer photoreceptors that express Rh1, and R7 and R8 are located above each other and express one of four Rh proteins. Rh3 and Rh4 are known to be expressed in R7. They showed that *defective proventriculus* (*dve*) is expressed in photoreceptors in pupal stages and blocks Rh3 expression. Interestingly, *orthodenticle* (*otd*) regulates *dve* expression but induces Rh3 in a *dve* mutant background. Hence, it was concluded that *otd*, which is upstream, induces *dve* and indirectly blocks Rh3 expression. Oorvashi Roy Puli (University of Dayton, Dayton, OH) presented another role of *dve* in an earlier time frame when Dorsal/Ventral (D/V) patterning is taking place in the eye. She found that loss of *dve* in the eye is causing dorsal eye enlargements. She also found that *dve* expression is localized to the dorsal eye half. She proposed that *dve* may be another dorsal eye fate selector. Abhishek Bhattacharya (Albert Einstein College of Medicine, New York) discussed a cross-reacting regulatory network between Class I and Class V helix loop helix (HLH) proteins that regulate neuronal differentiation. Extra macrochaetae (Emc) is a Class-V protein that inhibits Daughterless (Da), a Class-I protein, by the formation of inactive heterodimers. Loss of Emc results in premature differentiation, which leads to the formation of an ectopic morphogenetic furrow (MF), the position of which is dependent on *atonal* (*ato*), another HLH gene, expression. They concluded that Da induces Emc, which in turn blocks Da autoregulation in the MF. Carie M. Spratford (Indiana University, Bloomington) presented the role of Emc in regulating the Anterior/Posterior (A/P) and D/V patterning of the retina. Emc expresses ten rows behind the MF and is not dependent on the MF movement or initiation. Loss of *emc* leads to patterning defects. Emc also controls the rate of A/P patterning and establishes and maintains the D/V axis. Bonnie Weasner (Indiana University, Bloomington) showed that the Retinal Determination gene proteins, Sine oculis (So) and Eyes absent (Eya) form a complex, which is required for proper formation of the retina. In *so* and *eya* mutant retina, she observed increased levels of cell death and lack of cell proliferation. Interestingly, she found that *so* is responsive to Notch (N) signaling. When the N pathway is activated in *so* mutant eye discs, it results in the overgrowth of the eye-antennal disc. Mardelle R. Atkins (Baylor College of Medicine, Houston, TX) presented the role of Retinal Determination genes in the eye. The most upstream gene, *eyeless* (*ey*), is known to activate the expression of the downstream genes like *so, eya*, and *dachshund* (*dac*). While down-regulation of *ey* is required for differentiation and normal development of the eye initially, it is also required to specify eye fate. It was shown that *so* and *eya* ectopically repress anterior *ey* expression to promote eye formation. Cheng-Wei Wang (Academia Sinica, Taipei, Taiwan) provided an overview of the mechanism responsible for the subdivision of the monolayer epithelium into distinct eye and antennal fields. They showed that mutual antagonism between Cut, a homeodomain transcriptional repressor in the antennal field, and Ey in the eye field antagonize each other to restrict cell identity and thereby segregate eye and antennal fields. Sung-Yeon Park (Center for Biologics Evaluation and Research, Bethesda, MD) revealed the interaction between Decapentaplegic (Dpp) signaling and the JNK pathway in head capsule formation. The JNK pathway is associated with induction of apoptotic cell death caused by cell stress. It was observed that the JNK pathway was activated in *dpp* mutants that affect ventral head structures. It was concluded that the JNK pathway may play a dual role in Dpp-mediated head development, both as part of the normal morphogenesis and also through an apoptotic pathway when Dpp signaling is disrupted. Sang-Chul Nam (Baylor University, Waco, TX) presented data about *spastin* (*spas*), a gene that is required for rhabdomere elongation and photoreceptor morphogenesis. Mutation of *spas* causes neurodegenerative diseases. A mutation in this gene causes mislocalization of the apical domain and is reduced at the proximal section of the rhabdomere in the developing pupal eye. This mutant phenotype is similar to the previously described *crb* mutant and is suggestive of the fact that Spas is linked to the Crb pathway, playing a role in photoreceptor morphogenesis.

## TECHNIQUES AND TOOLS: FUNCTIONAL GENOMICS

One of the best features for research with *Drosophila* is the continual development and refinement of genetic and genomic tools and reagents available. Koen J. T. Venken (Baylor College of Medicine, Houston, TX) discussed the development of a Minos-mediated cassette exchange (MIMIC) system. This process involved the Minos transposon to create a gene trap cassette. It has the yellow dominant body marker and two inverted attP sites for recombinase-mediated cassette exchange (RMCE) for *in vivo* modification. Their initial results consisted of making 502 lines, of which 231 landed in genes, and 26% of those went into introns. They created multiple RMCE cassettes with EGFP, mCherry, and other smaller tags like Flag, Myc, or HA. This system had good insertions in multiple genes that showed correct small tag and fluorescent labeling. They are making thousands of these stocks that will ultimately be available at the Bloomington stock center.

Two independent groups are creating X-chromosome duplication stocks. Tom Kaufman's group is using overlapping BACs from the Pacman BAC clones of the X-chromosome. They are inserting these BAC clones on the 3rd chromosome. So far, they have 18 megabases of clones inserted, covering 80% of the X-chromosome euchromatin. By complementation analysis, one should be able to use these stocks to map a mutation on the X-chromosome down to a region of about 10 genes. These stocks are being submitted to the Bloomington stock center and the molecular data is found on Flybase. Kevin Cook (Bloomington *Drosophila* Stock Center) is also heading up a large-scale X-chromosome duplication project. These stocks are different from the Kaufman stocks in which the duplications are located on the end of the Y chromosome, allowing them to rescue lethality in mutant males. Therefore, these stocks can be used to map as well as rescue. They are producing these stocks through a series of genetic processes, including inversions, FRT recombinations, and so on. They have recovered 26 different inversions, which allows for 26 different nested deletions. This duplication set will be longer than the Kaufman set an average and complimentary to it.

Chemical mutation is a way to easily create saturable mutants and generate a lot of alleles; however, the mapping of those mutations is costly and time consuming. Therefore, those of us in the fly community that have mapped mutations were excited about the possibility that Rui Chen (Baylor College of Medicine, Houston, TX) presented. He demonstrated the ability and cost of using genomic capture sequencing to identify heterozygous mutations. The process he outlined started with the same rough mapping that is performed currently. This rough mapping will get the mutation down to roughly a megabase region. Then, next generation sequencing of that region can be performed. This sequencing technology can generate 6 gigabases of sequence per day per machine. This can roughly translate into a 30× coverage of the *Drosophila* genome costing about $3000. However, high-sequence coverage (at least 30×) is required to identify heterozygous mutations, especially SNPs. In an effort to reduce the cost, he performed padlock target capture sequencing, which uses a DNA synthesis process with homology arms around your target of interest to “tag” your sequence and isolate it for sequencing. Using this process, 285 amplicons were necessary for a 0.5-mbp region to identify mutations. The whole process, from start to finish, cost less than $1000 to map three separate mutants in that 0.5-mbp region.

## RNAi SCREENING

There are an increasing number of RNAi tools, stocks, and other resources becoming available. The Transgenic RNAi Resource Project (TRiP) has created thousands of UAS-driven RNAi lines that can be expressed by Gal4. However, as with all the other RNAi stocks currently available, the UAS construct in these stocks does not allow germline expression. Norbert Perrimon discussed a new RNAi vector that will be used in future TRiP stocks that will allow Gal4-induced expression in the germline as well. The FLIGHT RNAi tool (http://flight.icr.ac.uk/) is an online resource compiling data from many RNAi screens. FLIGHT is a new tool that helps in the visualization of a user's RNAi screen phenotype and also in the statistical analysis of RNAi screen results. Based on the database, off-target effects are reduced. It has text search tools for genes, mRNA, and cell lines. Its Batch RNAi search helps you batch download a text file of all results from all of the screens within the database for genes you are interested in. It also lets one add their RNAi screen data to the library for statistical analysis.

## IMAGING

The *Drosophila* embryo has multiple problems when trying to be visualized with current, standard confocal laser microscopy: phototoxicity, highly light scattering, and fast development. But, it is still very desirable to get confocal images from the whole embryo. Willy Supatto (California Institute of Technology, Pasadena) presented the first use of two-photon light sheet microscopy for *in toto* imaging of embryonic morphogenesis. This technique helps image and track every single cell in a developing organism or tissue, to digitalize and analyze all data in a standard and quantitative manner. The multifocal roster scanning microscopy helps scan several points at a time. However, this approach is still experimental and expensive.

## POSTER AWARD PRESENTATION

Jeremiah J. Zartman (Princeton University, Princeton, NJ) was the post-doctoral poster award winner. His winning poster was entitled, “Negative feedback bends the gene expression boundary in a developing tissue.” Hiroshi Ishimoto (Iowa University, Iowa City) was the second runner-up and Jianhua Huang (University of Maryland, College Park) was the third runner-up. W. Ryan Williamson (University of Texas Southwestern Medical Center, Dallas) won the Graduate Poster Award for his poster titled, “A dual function of the v-ATPase reveals a neuron-specific degradation pathway in *Drosophila*.” Vafa Bayat (Baylor College of Medicine, Houston, TX) was the second runner-up and Andrew D. Skora (Carnegie Institute of Science, Baltimore, MD) was the third runner-up. Cloyce E. Nelson (University of New Mexico, Albuquerque) won the Undergraduate Poster award for her poster entitled, “Deciphering *cis*-regulation in different adult muscle.” Cassandra Amesoli (New Mexico State University, Las Cruces) was the second runner-up and Alexander M. Tseng (University of Washington, Seattle) was the third runner-up.

The meeting proved to be a great success in providing a platform for presenting advances in wide spectrum of research areas in the *Drosophila* model. The credit for the success goes to Genetics Society of America, local organizers, and the fly community. This meeting further strengthened the widely accepted fact that the insect model of *Drosophila* holds immense promise for advancing biology, medicine, and human genetics. Finally, we look forward to the 52nd Annual *Drosophila* meeting at San Diego, CA, on March 30 to April 3, 2011.

